# Effekt eines mobilen Raumluftfilters auf die Aerosolbelastung in chirurgischen Untersuchungsräumen vor dem Hintergrund der COVID-19-Pandemie

**DOI:** 10.1007/s00113-021-00975-y

**Published:** 2021-02-26

**Authors:** Michael Oberst, Andreas Heinrich

**Affiliations:** 1 Klinik für Orthopädie, Unfall- und Wirbelsäulenchirurgie, Ostalb-Klinikum, Im Kälblesrain 1, 73430 Aalen, Deutschland; 2 Zentrum für Optische Technologien (ZOT), Hochschule Aalen, Beethovenstr. 1, 73430 Aalen, Deutschland

**Keywords:** COVID-19, Mobiler Raumluftfilter, SARS-CoV‑2, Übertragung, Aerosole, Untersuchungsraum, COVID 19, Indoor air cleaner, SARS-CoV‑2, Transmission, Aerosols, Examination room

## Abstract

Vor dem Hintergrund des Übertragungsweges von SARS-CoV-2-Viren durch kontaminierte Luftschwebeteilchen (Aerosole) wurde in einem chirurgischen Untersuchungsraum ohne Lüftungsmöglichkeit die Belastung an Luftschwebeteilchen mit bzw. ohne Verwendung eines Luftfiltergerätes gemessen. Hierbei zeigte sich bei Verwendung des Filtergerätes eine deutliche Reduktion der entsprechenden Luftpartikel. In Untersuchungs- und Behandlungsräumen, die baubedingt keine Lüftungsmöglichkeiten bieten, sollten mobile Luftfiltergeräte daher zum Einsatz kommen.

## Abstract (en)

Due to the airborne transmission of the coronavirus disease 2019 (COVID 19) via aerosols or microdroplets, this study investigated the effect of a mobile air filter system in a surgical examination room. The use of the air filter system led to a significant reduction of aerosols in the room. Therefore the use of a high efficiency air filtration device, in examination rooms with poor ventilation, e.g. lack of windows or local exhaust, is mandatory.

## Einleitung

Auch wenn die Übertragungswege von SARS-CoV‑2 noch nicht vollständig geklärt sind, spielt die Infektion durch viruskontaminierte Luftschwebeteilchen (Aerosole) offensichtlich die entscheidende Rolle bei der Infektion von Mensch zu Mensch [5, 6, 16]. In Abhängigkeit vom „Ausstoß“ (Sprechen, Atmen, Singen, Husten, Niesen …) werden Partikel unterschiedlichster Größe produziert und in die Umgebungsluft abgegeben. Während beim Atmen und Sprechen Partikelzellen von 0,75 bis 1,1 μm entstehen, werden beim Husten oder Niesen weitaus größere Teilchen ausgestoßen > 5 μm [2]. Je nach Größe der Teilchen ist die Verbreitung innerhalb eines Raumes unterschiedlich. Während Tröpfchen (>50 µm) relativ rasch zu Boden fallen, können Aerosole (<5 µm) über mehrere Stunden in der Raumluft nachgewiesen werden und auch problemlos aufgrund von Konvektion oder anderweitigen Luftströmungen Distanzen von mehreren Metern zurücklegen [4, 12, 15, 16]. Kommt es zu Inhalation, können diese Teilchen je nach ihrer Größe tief in den Respirationstrakt, bis auf die Ebene der Alveolen, vordringen [6, 10].

Definiert werden diese Imissionen („Feinstaub“) durch die Einheit PM_10_ („particulate matter“), welche 1987 von der US-amerikanischen Umweltschutzbehörde EPA (Environmental Protection Agency) als „National-Air-Quality Standard“ eingeführt worden war. Die Ziffer 10 steht in diesem Zusammenhang nicht für eine scharfe Trennung bei 10 µm des aerodynamischen Durchmessers, sondern soll das Abscheideverhalten der oberen Atemwege nachbilden: Partikel mit einem aerodynamischen Durchmesser von weniger als 1 µm werden vollständig einbezogen, bei größeren Partikeln wird ein gewisser Prozentsatz gewertet, der mit zunehmender Partikelgröße abnimmt und bei ca. 15 µm schließlich 0 % erreicht. Hierdurch leitet sich letztendlich auch die Bezeichnung PM_10_ ab, da bei ca. 10 µm genau die Hälfte der Partikel in die Gewichtung eingeht. Im Jahre 1997 wurde die Richtlinie um PM_2,5_ ergänzt, die dem lungengängigen (alveolengängigen) Feinstaub entspricht. Die Definition ist analog zu PM_10_, allerdings ist die Gewichtungsfunktion wesentlich steiler (100 %-Gewichtung < 0,5 µm; 0 %-Gewichtung > 3,5 µm; 50 %-Gewichtung bei ca. 2,5 µm). Eine weitere Erweiterung des Regelwerks ist durch PM_1_ gegeben, welche äquivalent zu PM_2,5_ für 1 µm definiert wird [1].

**Figure 1:**
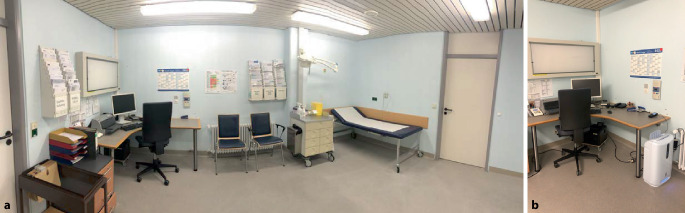

Vor dem Hintergrund der oben genannten Übertragungswege wurden bundesweit Vorsorgemaßnahmen propagiert bzw. angeordnet, mit dem Ziel, durch Verminderung der Aerosolexposition das Risiko der Übertragung von SARS-CoV‑2 zu reduzieren. Insbesondere in geschlossenen Räumen und Gebäuden soll hierbei zur Steigerung der Luftzirkulation regelmäßig gelüftet werden („AHA+L“-Regel [3]). Diese Maßnahme zur potenziellen Reduktion der an Aerosole gebundenen Viruslast ist in Räumen ohne Fenster allerdings nicht umsetzbar bzw. ohne größere baulich-technische Eingriffe in die Belüftungsanlagen kaum realisierbar. So ist beispielsweise im u.g. Untersuchungsraum der chirurgischen Klinik (Abb. 1) zwar eine wandmontierte Klimaanlage vorhanden, selbige funktioniert allerdings nach dem Umluftprinzip und hat daher keinerlei luftreinigende Wirkung, sondern trägt hingegen eher zur „Umwälzung“ der kontaminierten Teilchen bei und hat somit einen gegenteiligen Effekt.

Ziel der vorliegenden Untersuchung war es festzustellen, ob sich die nachweisbare Menge an Luftschwebeteilchen in einem Sprechstundenraum, der keinerlei Lüftungsmöglichkeiten bietet, durch Verwendung eines Raumluftfiltergerätes verringern bzw. die Konzentration an Schwebstoffen/Aerosolen in der Luft im Routinebetrieb einer chirurgischen Sprechstunde reduzieren lässt.

## Material und Methoden

Im Untersuchungsraum 2148 (Grundfläche 21 m^2^, Raumvolumen 52 m^3^, Abb. 1a) der Klinik für Orthopädie, Unfall- und Wirbelsäulenchirurgie des Ostalb-Klinikums in Aalen wurde am 12.11.2020 (Tag 2) ein Raumluftfiltergerät (Fa. DEMA-airtech, Stuttgart, Geräte-Typ AP-40) eingesetzt. Das Gerät besitzt laut Herstellerangaben eine maximale Filterkapazität von 320 m^3^ pro Stunde. Neben einem Aktivkohlefilter ist ein „High-efficiency-particulate-air“(HEPA)-Filter der Klasse H13 verbaut (European Norm 1822, mindestens Filtereffizienz von 0,3 µm/m^3^ pro h, Wirkungsgrad 99,95 %). Weiterhin wird eine Plasma- und UV-Licht-Bestrahlung der filtrierten Luft durchgeführt, was laut Herstellerangaben zusätzlich zur Filtereliminierung der Schwebstoffe, zu einer Abtötung von Viren und Bakterien in Höhe über 99 % führen soll (Guangdong Detection Centre of Microbiology, Report No. 2020SP8365R03E [8]). Das Gerät wurde im „Automatik-Modus“ betrieben, es stand in unmittelbar Nähe am Schreibtisch, zwischen Untersucher- und Patientenstühlen (Abb. 1a, b). Der Gehalt an Feinstaubpartikeln/Aerosolen der Raumluft wurde mittels eines auf dem Schreibtisch positionierten Messgerätes (IGERESS, Typ 6930, Fa. Shenzhenshi, Shenzen, China) gemessen. Hierbei wurden im Intervall von jeweils 15 Minuten die Anzahlen der Partikelgrößen PM_2,5_, PM_1,0_ und PM_10_ (jeweils in µg/m^3^) erfasst. Zum Vergleich war am Vortag (Tag 1) die Messung in derselben Art und Weise ohne die Verwendung des Luftfiltergerätes durchgeführt worden.

An den genannten Tagen wurde in dem Raum die Routinesprechstunde der Abteilung abgehalten. Diese bestreitet jeweils ein Facharzt der Klinik mit Unterstützung von 2 Sprechstundenhilfen. Gemäß den tagesaktuellen Bestimmungen der Kliniken Ostalb trugen alle im Raum befindlichen Personen einen Mund-Nase-Schutz, wobei diesbezüglich keine Filterklasse der Maske vorgegeben war.

## Ergebnisse

**Figure 2:**
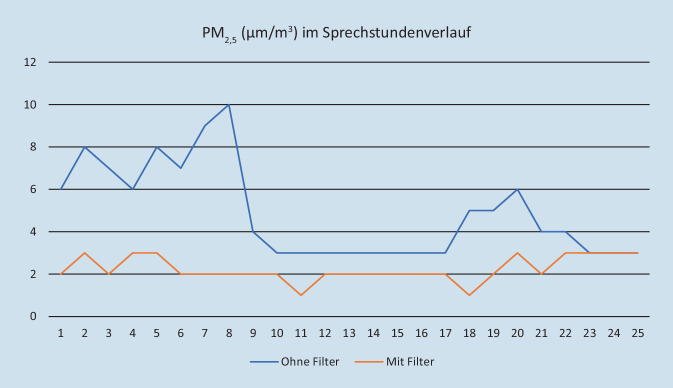

An den beiden genannten Tagen wurden 23 (Tag 1) bzw. 17 Patienten (Tag 2) in der Sprechstunde behandelt. Die Abb. 2 zeigt exemplarisch den Verlauf der Messkurve für die Teilchengröße PM_2,5_. (Die Kurven für PM_10_ und PM_1,0_ verlaufen jeweils annähernd identisch.)

In Tab. 1 und 2 sind die Werte für Mittelwert, Standardabweichung, Median und Interquartilenabstand (IQA) angegeben. Es zeigte sich am Tag 2 für alle genannten Teilchengrößen eine deutliche Reduktion der Werte im Vergleich zu Tag 1 um jeweils mindestens 50 %.

**Table 1:** 

	PM_1,0_	PM_2,5_	PM_10_
Mittelwert und Standardabweichung	3,36 ± 1,57	4,88 ± 2,16	5,12 ± 2,53
Median	3	4	4
1. Quartil	2	3	3
3. Quartil	4	6	6
IQA (3. Q–1. Q)	2	3	3

**Table 2:** 

	PM_1,0_	PM_2,5_	PM_10_
Mittelwert und Standardabweichung	1,24 ± 0,58	2,24 ± 0,59	2,24 ± 0,58
Median	1	2	2
1. Quartil	1	2	2
3. Quartil	2	3	3
IQA (3. Q–1. Q)	1	1	1

## Diskussion

Aufgrund des Fehlens einer kausalen Therapie für die COVID-19 und derzeit noch nicht flächendeckend verfügbarem Impfstoff gegen SARS-CoV‑2 ist die Vermeidung bzw. Verringerung der Aerosolexposition derzeit die wichtigste Maßnahme zur Infektionsprophylaxe. Bundesweit wird daher die „AHA+L“-Regel [3] propagiert bzw. behördlich angeordnet, wobei dem Tragen eines effektiven Mund-Nase-Schutzes eine sehr hohe Infektionsprävention zukommt [7, 13, 14]*.* Hinsichtlich der Effektivität des Luftaustausches im Raum (regelmäßiges Lüften) weisen neueste Untersuchungen darauf hin, dass in geschlossenen Räumen mit Publikumsverkehr und schlechter Ventilation das Risiko einer Infektion mit SARS-CoV‑2 erhöht ist [17]. Kähler et al. [11] konnten diesbezüglich im experimentellen Set-up unter Laborbedingungen nachweisen, dass mobile Raumluftfilter innerhalb sehr kurzer Zeit die Aerosolbelastung auf minimale Werte reduzieren können.

Die vorliegende Untersuchung konnte nun feststellen, dass durch die Verwendung eines Raumluftfilters die Menge an potenziell virusbelastenden Schwebstoffen in der Luft auch unter den Alltagsbedingungen einer chirurgischen Sprechstunde deutlich reduziert werden kann: Die Aerosolkonzentration reduzierte sich für alle erfassten Partikelgrößen um jeweils mindestens 50 % (vgl. Tab. 1 und 2 – auf eine Berechnung des Signifikanzniveaus haben wir an dieser Stelle verzichtet und nur rein deskriptiv Mittelwert, Standardabweichung und Median angegeben, da die Messreihen keine eindeutige Normalverteilung zeigten).

Die reale Sprechstundensituation ist allerdings gleichzeitig auch eine Schwäche der vorliegenden Untersuchung: Die Anzahl an Patienten an den beiden Untersuchungstagen waren nicht identisch, zudem wurde eine weitere Differenzierung (anwesende Begleitpersonen/konkrete Dauer des Aufenthalts im Untersuchungszimmer/mehrfacher Kontakt im Zimmer z. B. vor und nach dem Röntgen) nicht durchgeführt. Diese Inkonstanz im Versuchsaufbau ließe sich allerdings auch nicht durch eine exakt gleiche Verteilung der Sprechstundenkontakte umgehen – ist die Aerosolemission eines einzelnen Individuums doch von einer Vielzahl von Faktoren abhängig (Art und Dauer des Redens, Husten, Niesen, Bewegung im Raum etc.) und kann daher niemals standardisiert oder vorhergesagt werden. Vor diesem Hintergrund ist es auch nachvollziehbar, dass im vorliegenden Setting die Reduktion der Schwebeteilchen geringer war als unter den kontrollierten Bedingungen des Laborversuchs [11]. Dennoch sollte in künftigen Untersuchungen der zeitliche Verlauf der Partikelanzahl in Abhängigkeit vom Publikumsverkehr (Anzahl und Verweildauer der im Raum befindlichen Personen) berücksichtigt werden.

Ein anderer potenzieller Kritikpunkt der vorliegenden Untersuchung ist das verwendete Messgerät, welches hinsichtlich der Messqualität für die erhobenen Parameter zwar CE-zertifiziert, nicht aber DIN-geprüft ist. Die absoluten Werte der vorliegenden Untersuchung sind hierdurch mit einer möglichen Ungenauigkeit behaftet. Die Aussage der Untersuchung wird dadurch allerdings nicht geschwächt, da es sich – selbst wenn ein entsprechender Fehler vorliegen sollte – um einen durchgängigen, systematischer Fehler beider Messungen (mit/ohne Raumluftfilter) handeln würde. Selbiger ist irrelevant, da es in der vorliegenden Untersuchung nicht auf die absolute, sondern auf die relative Veränderung der Werte ankommt. So konnten wir eine deutliche Reduktion der Luftschwebeteilchen durch den Raumluftfilter nachweisen. Inwieweit sich hierdurch das konkrete individuelle Risiko einer potenziellen Infektion mit SARS-CoV‑2 tatsächlich reduziert, konnte in der vorliegenden Untersuchung selbstverständlich nicht überprüft werden. Allerdings darf konstatiert werden, dass bei einer mehr als hälftigen Reduktion der Virenmenge, der ein Individuum via Aerosolinhalation ausgesetzt ist, sich folgerichtig auch das Risiko der tatsächlichen Infektion um mehr als 50 % reduziert. Selbiges geschieht unabhängig von der individuellen Resistenzlage gegen das Virus. Auch andere Autoren raten daher zur Verwendung von Raumluftfiltern zur Verringerung des Infektionsrisikos mit SARS-CoV‑2 [15, 17].

Der verwendete Luftfilter kann bei maximaler Filterkapazität unter Volllast (320 m^3^/h) und einem Raumvolumen von 52 m^3^ den vom Umweltbundesamt [9] empfohlenen 6‑fachen Luftdurchsatz innerhalb einer Stunde gewährleisten. Weiterhin gibt der Hersteller an, durch UV-Licht und Plasmabestrahlung zusätzlich zur reinen Filterung der Aerosole auch eine über 99,9 %ige Eliminierung von Viren und Bakterien zu erreichen. Dies lässt eine weitere Reduktion der potenziell infektiösen Viruslast erwarten. Wie sich dies dann konkret auf die potenzielle Infektion mit SARS-CoV‑2 für das einzelne Individuum auswirkt, muss in weiteren Untersuchungen überprüft werden.

## Fazit für die Praxis

Durch die Verwendung eines mobilen Raumluftfilters kann die Aerosolbelastung innerhalb eines geschlossenen Raumes ohne Lüftungsmöglichkeit unter den realen Bedingungen einer chirurgischen Sprechstunde deutlich reduziert werden. Eine indirekte Minderung des potenziellen Infektionsrisikos einer durch Aerosole übertragenen Krankheit kann somit angenommen werden. In Untersuchungs- oder Besprechungsräumen von Krankenhäusern, die baubedingt keine Lüftungsmöglichkeiten bieten, ist der Einsatz von mobilen Raumluftfiltern unter den aktuellen Bedingungen der COVID-19-Pandemie aus unserer Sicht daher absolut sinnvoll.
